# Therapeutic approach to difficult-to-treat multidrug-resistant enterococcal infections

**DOI:** 10.1128/aac.01060-24

**Published:** 2025-09-11

**Authors:** Adrianna M. Turner, Paul Kinsella, William R. Miller, Glen P. Carter, Truc T. Tran, Benjamin P. Howden, Cesar A. Arias

**Affiliations:** 1 Department of Microbiology and Immunology, The University of Melbourne at the Peter Doherty Institute for Infection and Immunity, Melbourne, Australia; 2 Microbiology Department, Royal Melbourne Hospital, Melbourne, Australia; 3 Division of Infectious Diseases, Department of Medicine, Houston Methodist Hospital 23534 , Houston, Texas, USA; 4 Center for Infectious Diseases, Houston Methodist Research Institute 167626 , Houston, Texas, USA; 5 Department of Medicine, Weill Cornell Medical College 12295 , New York, New York, USA; 6 Department of Infectious Diseases and Immunology, Austin Health 3805, Heidelberg, Australia; 7 Microbiological Diagnostic Unit Public Health Laboratory, Department of Microbiology and Immunology, The University of Melbourne at the Peter Doherty Institute for Infection and Immunity, Melbourne, Australia; University of California San Francisco, San Francisco, California, USA

**Keywords:** *Enterococcus*, difficult-to-treat infection, vancomycin resistance

## Abstract

Difficult-to-treat (DTR) enterococcal infections, particularly those caused by multidrug-resistant *Enterococcus faecium* and *Enterococcus faecalis*, pose significant clinical challenges due to limited treatment options and high rates of treatment failure, compounded by a paucity of new antimicrobial agents in the development pipeline. Despite advances in understanding resistance mechanisms and *in vitro* synergistic antibiotic combinations, robust clinical data to guide therapy for severe or DTR enterococcal infections remain limited. This review synthesizes available evidence to inform optimal management strategies, including drug selection and dosing, while highlighting areas needing further research. Given the ongoing threat posed by multidrug-resistant enterococci, we emphasize the importance of gathering robust clinical data to guide best practices for managing these difficult-to-treat infections.

## INTRODUCTION

Healthcare-associated infections caused by multidrug-resistant (MDR) bacteria are a critical public health threat associated with high rates of treatment failure, morbidity, and mortality. Enterococci are commensals of the human gastrointestinal tract (GIT) that have emerged as leading causes of nosocomial infections, particularly in critically ill and immunocompromised patients (1). Enterococci have been implicated in a diverse range of infections, but bloodstream infections, endocarditis, intra-abdominal, and urinary infections are the most commonly seen, often requiring aggressive targeted therapy.

One of the most challenging issues that clinicians encounter when faced with an invasive enterococcal infection is the limited choice of antimicrobial agents since many hospital-associated isolates (e.g., *Enterococcus faecium*) are resistant to most anti-enterococcal antibiotics. Indeed, resistance to aminopenicillins, vancomycin, daptomycin, and linezolid is now reported among nosocomial isolates of *E. faecium*, leaving extremely limited alternatives for treatment. Moreover, recent studies on the epidemiology of enterococcal bacteremia indicate that failure to eradicate the bloodstream infection early in the course of the disease is associated with increased likelihood of death (2), emphasizing the fact that successful antimicrobial therapy is key for patient survival, particularly in those with immunocompromised conditions or with multiple co-morbidities.

Another major issue when dealing with enterococcal infections is the paucity of clinical data supporting a particular treatment. Despite the increasing number of patients infected by these organisms, robust clinical data to guide therapy in severe enterococcal infections are lacking. Here, we provide an updated overview (2022–2024) of the therapeutic options and approaches for drug optimization in difficult-to-treat (DTR) vancomycin-resistant enterococcal (VRE) infections. Although low-level vancomycin resistance is intrinsic to some *Enterococcus* species beyond *Enterococcus faecalis* and *E. faecium*, this review will focus on the latter two species, as they are the most frequently encountered in clinical infections.

## ENTEROCOCCI IN THE HEALTHCARE SETTING

Enterococci can survive for long periods on environmental surfaces like medical equipment, bed rails, and doorknobs (3). They are tolerant to different temperatures, chlorine, and some alcohol preparations (4, 5), likely contributing to their success in the hospital setting. *E. faecalis* is the most common species isolated from human infections, particularly those of community-onset. However, in the last two decades, a major increase in hospital-associated infections caused by *E. faecium* has occurred, particularly in specific patient populations such as those with hematological malignancies, bone marrow, and liver transplants (6). Although the rise in *E. faecium* infections is likely multifactorial and remains under investigation, epidemiological studies suggest that certain problematic lineages of *E. faecium* are particularly well-adapted to the hospital environment (7, 8). These strains are more likely to acquire resistance to clinically relevant antimicrobials and possess survival advantages in the GIT, potentially due to other protective mechanisms such as bacteriocin production (9). Transmission of VRE has been documented through direct contact with colonized or infected patients, or by indirect contact via contaminated environmental surfaces or the hands of healthcare workers (4). Risk factors for VRE nosocomial transmission include prolonged hospitalization, use of broad-spectrum antibiotics, surgery, immunocompromised state, or occupying a room with a previously infected or colonized patient, among others (10). Indeed, one of the intrinsic characteristics of enterococci is their tolerance to antimicrobial agents that are often used as empiric therapy in hospitalized patients (e.g., β-lactams, vancomycin). This resistance phenotype promotes increased colonization of the GIT, which seems to be associated with an increased likelihood of developing an infection and increases the risk of transmission. Indeed, reported colonization rates vary by population type from 16.2% in solid organ transplant to up to 20% in hematopoietic cell transplant (11, 12). Infection rates are much higher in colonized patients compared to those who are not colonized (relative risk 24.15 [95% CI = 10.27–56.79]) (12). Active surveillance, contact precautions for colonized and infected individuals, strict hand hygiene practices for healthcare workers, and judicious use of antimicrobial agents can effectively curtail VRE transmission chains. More recently, microbiome manipulation to reduce VRE in the GIT is a strategy that is gaining traction to reduce the risk of infection and, possibly, transmission of VRE (13).

## DIFFICULT-TO-TREAT MULTIDRUG-RESISTANT ENTEROCOCCAL INFECTIONS

There is no agreed universal definition of what constitutes a DTR enterococcal infection. For ease of discussion, here we are using the term “difficult-to-treat” to include infections that are either “persistent” (failure to eradicate the organism despite seemingly appropriate antimicrobial therapy) or “recurrent” (defined as patients who had clearance of the bacteremia during hospitalization, with a second positive culture also during this period) (2). While these terms, persistence and recurrence, describe microbiological outcomes following an adequate course of treatment, we use the term DTR to capture a broader concept encompassing not only the microbiological characteristics of the infecting strains but also the clinical context, including patient-specific factors and antimicrobial treatment challenges, as outlined below. Consequently, DTR clinical scenarios typically exclude patients who are not significantly immunocompromised, have modifiable sources of infection, are not at risk for GIT colonization and/or selection of MDR enterococci, or are infected with organisms that remain broadly susceptible to available antimicrobial agents. Although these definitions are not standard, they capture the spectrum of challenging enterococcal infections encountered during routine clinical practice.

A cohort study of patients with enterococcal bacteremia (VENOUS) by Contreras et al. examined several outcomes, including lack of clearance of the organisms on day 4 while receiving at least 48 hours of active anti-enterococcal therapy (described as “microbiological failure”) (2). The cut-off of 4 days was derived from a previous study in bloodstream infections that suggested that day 4 of bacteremia was associated with increased mortality (14). Based on the VENOUS cohort, we recently (15) defined recurrent bacteremia as patients who had clearance of the bacteremia during hospitalization, with a second positive culture also during this period, or two negative cultures at least 1 day apart, followed by at least one positive culture. Overall, persistence and recurrence, described as recalcitrant bacteremia in this study, occurred in 3.2% of patients, although reports suggest that up to 12% of patients may exhibit this phenotype (16).

Clinically, DTR bacteremia is influenced by many factors, including source control of deep-seated infections (17), presence of infected central venous access devices, and the pharmacokinetics/pharmacodynamics (PK/PD) of the antimicrobial agents used against enterococci, among others. It is important to note that these DTR infections are often reported in patients with important degrees of immunosuppression who have been on antimicrobial therapies for a prolonged period of time and, often, associated with “domination” of the GIT microbiome by enterococci (18, 19). Historically, endocarditis is a disease that often leads to persistent bacteremia. *E. faecalis* is, by far, the most common cause of enterococcal endocarditis, and cases of multidrug-resistant *E. faecium* are rare but still occur (20). Indeed, in a recent multicenter prospective observational cohort study of enterococcal endocarditis in Spain, 7.0% of cases were attributed to *E. faecium* (21). However, it is unclear what proportion of these organisms were multidrug-resistant. In one of the largest cohort studies (*n* = 244 cases) to examine persistent enterococcal bacteremia, Bussini et al. demonstrated a higher mortality among patients with persistent enterococcal bacteremia than without (defined as positive blood cultures at least 72 hours after commencement of antibiotics) (22).

There are limited therapeutic options for the treatment of VRE bacteremia, as well as a paucity of new antimicrobial agents in the development pipeline. In the next sections, we describe the recent evidence for available treatment options for VRE infections, particularly focused on the importance of dose optimization and the emerging role of combination therapies.

### Daptomycin and combinations

#### Mechanisms of action and resistance

Daptomycin (DAP) is a cyclic lipopeptide that was first approved for clinical use in the United States (2003) for complicated skin and skin structure infections (cSSTIs) (4 mg/kg of body weight) and later for bacteremia and right-sided endocarditis caused by *Staphylococcus aureus* (6 mg/kg). DAP has a distinct mechanism of action that disrupts cell membrane and cell wall homeostasis by forming a tripartite complex with phosphatidylglycerol and undecaprenyl-coupled intermediates (lipid II) at the division septum (23). The antibiotic also appears to delocalize important peripheral proteins involved in both peptidoglycan and phospholipid metabolism (23). DAP does not have a specific FDA-approved indication for infections caused by VRE but is often used “off-label” for this indication due to the potent *in vitro* bactericidal activity against these organisms. The European Committee on Antimicrobial Susceptibility Testing (EUCAST) does not provide breakpoints due to pharmacological (PK) evidence suggesting that high-dose DAP therapy (10–12 mg/kg/day) may be suboptimal to treat infections caused by isolates at the upper end of the wild-type distributions for *E. faecalis* (MIC 4 mg/L) or *E. faecium* (MIC 4 or 8 mg/L) (24). The Clinical and Laboratory Standards Institute (CLSI) breakpoints address this issue with two sets of breakpoints, one for *E. faecium* (susceptible dose dependent at ≤4 mg/L and resistant at MICs of ≥8 mg/L) and another for other enterococcal species (susceptible ≤2 mg/L, intermediate at 4 mg/L, and resistant at MICs of ≥8 mg/L) (25, 26).

DAP resistance is most commonly associated with two distinct sets of mutations involving cell envelope stress response systems (such as *liaFSR, walKR,* and *madRS*) and phospholipid biosynthetic pathways (*cls, gdpD*, *dak*, and *cfa*) (27, 28), and more recently, mutations in *rpoB* (29). Evolutionary selection experiments have shown (30) that mutations in genes encoding proteins that function in cell envelope stress signaling occur first (i.e., Ile177del LiaF in *E. faecalis*; W73C LiaR and T120A LiaS in *E. faecium*), followed most frequently by mutations in genes encoding enzymes involved in phospholipid metabolism, including the gene coding for cardiolipin synthase *cls*, leading to a fully resistant phenotype (28, 31). Importantly, changes in LiaFSR have been associated with DAP tolerance, or impaired antibiotic killing *in vitro*, and this phenomenon is postulated to contribute to enterococcal survival and the emergence of resistance at lower drug exposures (32). Furthermore, DAP bears similarities to several antimicrobial peptides of the innate immune system (33). Thus, exposure to the host environment may prime the pathways involved in resistance. Additionally, resistance has been documented to develop during the course of DAP therapy, particularly in immunocompromised patients and during severe invasive infections, with prior DAP exposure linked to the risk of isolating a subsequent DAP-resistant isolate (34). An important consideration is that the development of resistance to DAP, particularly via the LiaFSR system, is associated with increased susceptibility to β-lactams, a phenomenon designated as the seesaw effect (35, 36) whose mechanistic bases are still unknown. Both *ex vivo* PK/PD models and *in vivo* studies support using the synergistic interaction between DAP and β-lactams as a treatment strategy for high-inoculum infections caused by DAP-tolerant or -resistant strains (see below).

#### Clinical use of DAP monotherapy

The body of evidence suggests that the FDA-approved dose of DAP for *S. aureus* bacteremia (6 mg/kg/day) is insufficient to treat invasive enterococcal infections (37). Indeed, an analysis of the mutant prevention concentration for DAP dosages between 4 and 12 mg/kg/day found that the previously recommended FDA doses at 4 to 6 mg/kg/day fell into a mutant selection window, suggesting the risk of developing resistance was increased, especially in severe infections where the concentration of DAP is lower at infection sites due, in part, to the high protein binding of the antibiotic (38). The notion that doses of ≥10 mg/kg are needed for therapeutic efficacy is also supported by several *in vitro* PK/PD studies (32). In a recent multi-center prospective study, patients who received higher doses of DAP (≥11 mg/kg) had lower mortality than patients who received lower doses (8 to <11 mg/kg) (adjusted OR = 0.85; 95% CI = 0.73–0.99; *P* = 0.03), although higher doses were associated (*P* = 0.04) with an increased frequency of elevated creatine kinase (>2,000 U/L; 3.9%) (37). Similarly, lower doses of DAP (<8 mg/kg/day) were associated with higher rates of patient mortality in post-liver transplant patients with VRE bloodstream infection (BSI) (<60 days after transplantation) (*P* = 0.024) (39). Thus, *in vitro*, animal, and clinical data support the use of higher doses of DAP for the treatment of VRE BSI isolates with susceptible DAP MICs.

#### Combination of DAP plus other antibiotics

Due to the propensity of enterococci to develop resistance during therapy and the ability of these organisms to become tolerant to DAP after their cell envelope stress responses are activated by DAP and antimicrobial peptides produced by the innate immune system, there is an argument that DAP monotherapy (even at high doses) may still be inadequate to treat severe enterococcal infections, especially high inoculum infections (e.g., infective endocarditis [IE]) (40). Enterococci are intrinsically resistant to most β-lactams (particularly cephalosporins) due, at least in part, to the presence of a low-affinity class B penicillin-binding protein (PBP-4 in *E. faecalis*, PBP-5 in *E. faecium*) encoded in their core genome. Nonetheless, development of DAP resistance is associated with increased susceptibility to β-lactams (the see-saw effect), an observation that is supported by *in vitro* and *in vivo* data (35). While the specific mechanisms underlying the “see-saw” effect have yet to be fully elucidated, there is evidence that activation of the LiaFSR system and downstream proteins such as LiaX play a major role in the phenotype (36). LiaX exhibits a dual sensing and regulatory activity in the activation of the LiaFSR system and has been shown to be capable of binding both DAP and PBP-5 from *E. faecium* (41, 42). On the other hand, some studies have shown that exposure to β-lactams reduces the net positive bacterial surface charge and leads to an increase in DAP binding (43).

Using a PK/PD model of infective endocarditis, the combinations of DAP plus ampicillin, ertapenem, or ceftaroline exhibited enhanced efficacy against *E. faecium* isolates with LiaFSR substitutions (which are likely predisposed to develop DAP resistance) compared to DAP monotherapy, even at higher doses (44). Additionally, DAP plus ampicillin was effective against all strains, and no development of resistance was observed (44). This enhanced activity was also evident when using a DAP-resistant *E. faecium* strain (35). Using an *in vivo* rat model of IE, the most reliable combination was that of DAP plus ampicillin, showing a significant decrease in bacterial burden in vegetations compared to controls (35, 40). Nevertheless, it remains a challenge to demonstrate that the synergistic effect observed between DAP and β-lactam antimicrobials translates into improved clinical outcomes. A relatively recent multi-cohort study suggested that DAP plus β-lactam treatment was significantly associated with a higher rate of clinical success than DAP alone for treatment of VRE BSIs (adjusted OR = 3.19; 95% CI = 1.61–6.33; *P* = 0.001) (Table 1) (45). Furthermore, several case reports (46, 47) have described the successful use of the combination of DAP plus β-lactam, including in patients with difficult-to-treat VRE joint infection, bacteremia, and infective endocarditis. Of note, the combination showed synergism despite the fact that the isolates were resistant to ampicillin and had increased MICs for DAP.

**TABLE 1:** Emerging evidence (from 2022) for DAP combination therapies for VRE infections^*a*^

Combination antimicrobial(s)	Design	Dose/median dose	Conclusion	Reference
β-lactam *Carbapenem, Penicillin, Cephalosporin*	Observational multi-cohort (*n* = 430 patients; DAP, *n* = 45; DAP+BL, *n* = 385)	Daptomycin: mean 10 mg/kg	Daptomycin + β-lactam had a higher clinical success rate than daptomycin alone, in those with MIC <2	Chuang et al. (45)
β-lactam *Carbapenem, Penicillin*	Case report	Daptomycin: 10 mg/kg Meropenem, ertapenem, ampicillin: various doses	Successful treatment of a bone and joint infection caused by VRE using high-dose daptomycin plus β-lactams	La et al*.* (46)
Fosfomycin	Prospective cohort (*n* = 106 patients)	Daptomycin: median 10.18 mg/kg (IQR 9.43–10.70) Fosfomycin: 16 g/day (IQR 8–22.5)	Higher daptomycin doses and fosfomycin susceptibility were independently associated with lower mortality in patients with VRE BSI	Chuang et al. (48)
Fosfomycin	Retrospective cohort (*n* = 224 patients; DAP, *n* = 176; DAP+fosfomycin, *n* = 48)	Daptomycin: median 9.8 mg/kg (IQR 9.0–10.4) Fosfomycin: median 12 g/day (IQR 6–21)	The combination of high-dose daptomycin therapy with fosfomycin improved the survival rate in patients with VRE BSI	Tseng et al. (49)
Chloramphenicol	Case report	Daptomycin: 7–10 mg/kg Chloramphenicol: 12.5 mg/kg	First case of a VRE endocarditis infection that was successfully managed with daptomycin and chloramphenicol combination therapy	Shah et al. (50)
Phage Φ9184 or ΦHi3	Case report	Daptomycin dose not reported Φ9184: 1 × 10^9^ plaque-forming units ΦHi3: 1 × 10^9^ plaque-forming units	A case of recurrent VRE BSI for 7 years, which failed antibiotics. Bacteremia resolved 24 hours after Φ9184 administration combined with daptomycin, but recurrence occurred after 31 days. The patient remained VRE BSI-free after the addition of 2 phages and daptomycin	Stellfox et al. (51)

^*a*^ DAP, daptomycin; BL, β-lactam; BSI, bloodstream infection; IE, infective endocarditis; IQR, interquartile range.

Fosfomycin, an antibiotic that interferes with early stages of peptidoglycan synthesis, has synergism with DAP against methicillin-resistant *Staphylococcus aureus* (MRSA) in a rabbit model of endocarditis (52) and showed mixed results in a randomized trial as compared to DAP monotherapy in patients with MRSA BSI (53). Fosfomycin inhibits the MurA enzyme, which catalyzes the first committed step in peptidoglycan biosynthesis, and the mechanism of the synergistic relationship between DAP and fosfomycin is thought to be similar to that of β-lactam combinations. DAP in combination with fosfomycin has only been shown to be synergistic against VRE *in vitro*. Two separate patient cohort studies comparing DAP monotherapy versus DAP combination therapy with fosfomycin for treatment of VRE BSI indicated that combination therapy was associated with better clinical outcomes through associations with lower patient mortality. Both analyses indicated the combination was less effective for patients with fosfomycin-resistant isolates (MICs > 64 mg/L), with the highest fosfomycin MICs associated with increased mortality (48, 49). Furthermore, hypokalemia, a common adverse event associated with the use of fosfomycin, was frequently reported, which could be related to the interactions of multiple drugs administered to critically ill patients and limit the utility of this combination. Thus, further clinical data supporting the use of DAP with β-lactam and other antibiotics that interfere with peptidoglycan homeostasis are needed.

### Linezolid and other oxazolidinones

Linezolid is an oxazolidinone with bacteriostatic activity against Gram-positive bacteria, including *E. faecium* and *E. faecalis*. Oxazolidinone antimicrobials (linezolid, tedizolid, and contezolid) bind to the 50S ribosomal subunit and disrupt protein synthesis by altering the position of the aminoacyl moiety of the aa-tRNA. Linezolid was first approved by the FDA in 2000 (600 mg every 12 hours) and remains the only approved antimicrobial for VRE BSIs. Resistance to linezolid is reported globally in enterococci and is often mediated through mutations in genes encoding the 23S rRNA involving all alleles (four alleles in *E. faecalis*, six alleles in *E. faecium*) (54). Of note, recombination between alleles can rapidly lead to the replacement of linezolid-susceptible 23S rRNA genes with the resistant allele (55). Linezolid resistance in VRE is also associated with the presence of several transferable resistance genes, including *cfr*, *poxtA*, and *optrA*, encoded within mobile transposons or plasmids (56). The Cfr enzyme methylates the C8 of adenosine at position 2,503 of the 23S rRNA, and *poxtA* and *optrA* are thought to mediate oxazolidinone resistance by protecting the ribosome, preventing binding of the antibiotic (57, 58).

The clinical breakpoint for linezolid susceptibility in *Enterococcus* spp. differs between CLSI and EUCAST guidelines, with CLSI reporting isolates with a MIC ≤2 mg/L susceptible and a MIC ≤4 mg/L intermediate. There is no intermediate classification in EUCAST, with all isolates with a MIC ≤4 mg/L deemed susceptible to the antibiotic. However, both CLSI and EUCAST consider linezolid resistance in *Enterococcus* spp. isolates with an MIC ≥8 mg/L. High levels of variation in linezolid plasma concentrations are reported in patients with normal to augmented renal function, the elderly, and those with critical illnesses (59). Clinical data from the compassionate use of linezolid suggested that a free drug time above the area under the curve to MIC ratio (*f*AUC_24_/MIC) of >100 correlated with successful treatment outcomes (59). Using this *f*AUC_24_/MIC target, one study concluded that the standard linezolid dose (600 mg every 12 hours) may result in insufficient exposure to effectively treat VRE isolates with higher, but susceptible, MICs (~50% probability of target attainment for MIC 2 mg/L) (60, 61). In a separate multicenter observational study, there was a significant association with linezolid dose per body weight and body height and patient mortality (adjusted OR = 0.02; 95% CI = 0.002–0.23; *P* = 0.002) (62). However, the mean calculated *f*AUC_24_/MIC was no different between the groups (213 versus 233, *P* = 0.42), and linezolid MIC up to 4 mg/L was not associated with patient mortality (*P* = 0.95) (62). Increased linezolid dosing also enhances the risk of hematologic toxicity (thrombocytopenia). Thus, additional studies are needed to clearly define the therapeutic target and role of linezolid dose adjustment in serious VRE infections.

Several studies have examined risk factors for clinical failure or the emergence of resistance to linezolid. In an 11-year retrospective cohort study (2007–2018) of patients who failed linezolid therapy for VRE BSI, early initiation of linezolid treatment within the first 48 hours of bacteremia (as compared to no VRE active therapy) was a protective factor from mortality in the univariate analysis (*P* < 0.001), but not in multivariate analyses (*P* = 0.088) (63). However, these results were not substantiated in a prospective 3-year, case-control study (2019–2022) of linezolid-resistant VRE infections in a region where linezolid use is high (64). Acquisition of a linezolid-resistant isolate was significantly associated with prior use of linezolid (OR = 10.13; 95% CI = 4.13–24.82; *P* ≤ 0.001) and carbapenems (OR = 2.85; 95% CI = 1.62–5.02; *P* ≤ 0.001), with linezolid-resistant isolates significantly associated with increased patient mortality (*P* = 0.003). Linezolid-resistant isolates were more likely to be resistant to several antibiotic classes, such as vancomycin, with some strains being only susceptible to DAP. The different outcomes between these two case series could be related to the time at which the isolates were collected (2007–2018 versus 2019–2022) since resistance to linezolid is reported to be increasing in VRE as linezolid consumption has increased (65). In a large retrospective cohort of patients with enterococcal bacteremia in the Veterans Affairs system in the US, linezolid appeared to perform worse than DAP (increased 30-day mortality; RR, 1.13; 95% CI = 1.02 to 1.26; *P* = 0.015), even in patients who were started on linezolid and later switched to DAP (66, 67). Thus, these data suggest that linezolid may not be the most effective antibiotic in DTR infections due to enterococci.

Tedizolid is approved in the United States (2014) and European Union (2015) for the treatment of acute bacterial skin and skin structure infections (ABSSSIs) caused by susceptible Gram-positive bacteria. Tedizolid exhibits higher potency than linezolid and retains lower MICs against linezolid-resistant *Enterococcus* spp. and *Staphylococcus* spp. Moreover, tedizolid seems to be active *in vivo* against *S. aureus* strains carrying the *cfr* gene (68, 69). There is some concern as to whether the lower MIC in enterococci translates to efficacy, as tedizolid was found to be inferior to either DAP or linezolid against one *cfr*(B) positive isolate of *E. faecium* in a mouse peritonitis model (70). CLSI has established a clinical breakpoint for tedizolid only for *E. faecalis*, defining susceptibility at an MIC ≤0.5 µg/mL, while EUCAST has not endorsed a specific breakpoint for tedizolid (71, 72). It is worth noting that tedizolid and linezolid differ significantly in their dosing regimens and resulting drug exposures. Tedizolid, when administered orally as a once-daily 200 mg dose, results in an AUC_0-24_ of 25.6 ± 8.4 µg⋅h/mL (73). In comparison, oral linezolid is dosed at 600 mg twice daily, yielding an AUC_0-τ_ of 138 ± 42.1 µg⋅h/mL (74, 75).

Contezolid is in late-stage clinical development and is only approved by the National Medical Products Administration in China for the treatment of complicated skin and soft tissue infections in susceptible bacteria (76). In the United States, contezolid is undergoing clinical development for the treatment of ABSSSIs and diabetic foot infections. Preliminary data from a Phase III trial in China suggest that contezolid shows a lower tendency to depress platelet counts than linezolid, indicating it could act as an alternative treatment for patients who are unable to tolerate linezolid. There are no breakpoints available for contezolid from either CLSI or EUCAST for *Enterococcus* spp. but preliminary epidemiological cut-off values have been proposed for *E. faecalis* and *E. faecium* at a MIC >4 mg/L (77).

Due to the difficulties in treating DTR enterococcal infections, there is a reasonable consideration to use linezolid as part of a combination regimen. However, most *in vitro* studies do not support an additional benefit of linezolid combined with other anti-enterococcal antibiotics (no evidence of synergism or antagonism). Synergism between linezolid and fosfomycin has been demonstrated *in vitro*, with the combination of fosfomycin and linezolid resulting in a synergistic or additive effect in a panel of clinical enterococcal isolates (68.4% or *n* = 13/19) (78). The synergism was present in fosfomycin-resistant strains *in vitro*, with high concentration fosfomycin (2,048 or 4,096 mg/L) resulting in a significant decrease (2 × log_10_ CFU/mL) during *in vitro* time kill experiments. However, there was limited data on the species of enterococci, the presence of vancomycin resistance, or the mechanism of fosfomycin resistance in the strain collection tested. The synergism between linezolid and fosfomycin was further tested against vancomycin-resistant *E. faecium* biofilms, with higher concentrations of linezolid (2 or 4× MIC) and fosfomycin (1× MIC) resulting in a significant decrease in biofilm biomass (79). These studies indicate that more experimental data are needed to investigate the potential synergistic effect between linezolid and other antimicrobials for VRE infections. Of note, linezolid has been added to other antibiotics to increase anti-enterococcal activity in certain circumstances of “rescue” of DTR enterococcal infections, independently of the synergistic effect. This approach has not been validated, and there is a paucity of clinical data to support this practice.

### Newer tetracyclines (tigecycline, eravacycline, and omadacycline)

Tigecycline is a semisynthetic glycylcycline that has enhanced binding to the 16S rRNA, which can evade some tetracycline resistance mechanisms mediated through the *tet* resistance genes. Tigecycline is approved for the treatment of cSSTIs, complicated intra-abdominal infections, and community-acquired pneumonia caused by susceptible Gram-negative or Gram-positive bacteria and has emerged as a “last-resort” antibiotic for VRE infections due to activity *in vitro* against multidrug-resistant enterococci. Resistance to tigecycline is considered low worldwide in both *E. faecium* (1%) and *E. faecalis* (0.3%), with resistance occurring through mutations in the ribosomal protection protein S10 (above) and amplification of tetracycline resistance genes *tet*(L) and *tet*(M). Tigecycline resistance has been recently described to emerge after exposure to the drug from a patient with a BSI caused by a linezolid- and vancomycin-resistant *E. faecium* strain through two amino acid deletions (K57 and Y58) in the S10 protein (encoded by the *rpsJ* gene) and a deletion in the *tet*(M) leader peptide, suggesting resistance can emerge *de novo* (80).

Eravacycline is a novel fluorocycline approved for treating complicated intra-abdominal infections caused by Gram-negative and Gram-positive bacteria. EUCAST breakpoints for eravacycline for both *E. faecalis* and *E. faecium* include susceptible (≤0.125 mg/L) and resistant (>0.125 mg/L) categories. However, the FDA breakpoint has a susceptible-only category of ≤0.06 mg/L for both species. An *in vitro* analysis of eravacycline activity against enterococci isolated from the Chinese CHINET study suggested robust activity against *E. faecalis* (97%–99.5% susceptible) and *E. faecium* (88.2%–94.4% susceptible) (81). Among the vancomycin-resistant *E. faecium* isolates, susceptibility rates were slightly lower (76.7%–90%) (81). A similar analysis using global enterococcal isolates collected from 2017 to 2020 indicated that eravacycline was highly active against *E. faecalis* (*n* = 1,876) and *E. faecium* (*n* = 1,724) (MIC_90_ 0.06 mg/L for both), including vancomycin-resistant isolates (98.3% susceptible by EUCAST and 76.5% susceptible by CLSI) (82). Eravacycline is generally active against enterococcal isolates harboring the acquired *tet* [*tet*(M), *tet*(K), *tet*(L), *tet*(A), and *tet*(B)] resistance genes, although expansion in *tet* gene copy number in tandem with *rpsJ* mutations (encoding the ribosomal S10 protein) has been associated with resistance to both tigecycline and eravacycline (83).

Omadacycline is an aminomethylcycline antibiotic that has an FDA-approved indication for ABSSSI caused by susceptible bacteria (includes *E. faecalis* but not *E. faecium*) and also in community-associated bacterial pneumonia. This antibiotic is available in both oral and intravenous formulations. The FDA has established a susceptibility breakpoint for omadacycline at ≤0.25 µg/mL for *E. faecalis*, without differentiating based on vancomycin resistance status (84). Omadacycline has potent *in vitro* activity against *E. faecium*, with MIC_50_ and MIC_90_ of 0.06 and 0.12 µg/mL, respectively (>1,500 *E*. *faecium* strains, including 93.8% VRE and 93.7% harboring Tet determinants [efflux or ribosomal protection]) (85). In an *in vivo* murine model of peritonitis, omadacycline was highly active against multidrug-resistant *E. faecium* strains, including vancomycin and ampicillin-resistant and DAP-resistant, yielding significantly higher survival in animals compared to vancomycin and DAP (85).

One of the main limitations of the newer tetracyclines is that these compounds do not reach sufficient concentrations in blood to be considered agents of choice for enterococcal bacteremia. However, these compounds achieve high tissue concentrations, particularly in the biliary tract, making them useful in the treatment of intra-abdominal infections since their spectrum of activity also targets multidrug-resistant Enterobacterales, which are common pathogens in these infections. This pharmacological property and robust *in vitro* activity make them attractive as part of a combination therapy with agents that are optimal for bacteremia (e.g., DAP or the combination of DAP plus ampicillin) when the focus of infection involves the GI or biliary tract. Some case reports, including one with endocarditis treated with DAP plus tigecycline (86), seem to support this approach, but more robust clinical data are needed.

### Oritavancin

Lipoglycopeptides (dalbavancin and oritavancin) are long-lasting, semisynthetic antimicrobials with potent activity against Gram-positive bacterial pathogens. Unlike dalbavancin, oritavancin has *in vitro* activity against both *vanA* and *vanB* VRE. Oritavancin inhibits peptidoglycan synthesis by binding to peptidoglycan precursors, and the mechanism of action involves additional points of contact on peptidoglycan stem peptide compared to vancomycin (87). Additionally, part of the oritavancin molecule also interacts with the membrane, likely increasing its affinity for peptidoglycan precursors and altering membrane homeostasis (88). Oritavancin has a half-life of ~393 hours, which allows for weekly administration in ABSSIs. Surveillance studies in the United States (89) and Europe (90) over 2010–2019 indicated that enterococcal isolates have remained susceptible (MIC < 0.5 mg/L) during the sampling timeframe, including vancomycin- and DAP-resistant strains. Although data are limited, preliminary studies evaluating oritavancin for the treatment of invasive enterococcal infections appear promising. A single case report suggested 1,200 mg of oritavancin for 6 weeks (with a more aggressive administration schedule) was successful for treating liver abscesses caused by linezolid- and vancomycin-resistant *E. faecium* (*vanA*, *optrA*, and *cfrD* genotype) (91). A retrospective cohort (92) and a case report (93) studied the potential for oritavancin use after BSIs or IEs treatment with standard-of-care antibiotics. Sequential therapy with oritavancin was associated with earlier discharge of patients (94%) and institutional cost avoidance (92), suggesting oritavancin could be effective as sequential antimicrobial treatment for BSIs caused by enterococci. Moreover, *in vitro* data suggest that oritavancin exposure can also lead to the see-saw effect and is likely to be synergistic with β-lactams (94), opening novel strategies for treatment. However, clinical data to support these assumptions are still lacking.

### Teicoplanin for vanB-carrying enterococcal infections

Teicoplanin is a bactericidal glycopeptide antibiotic with a mechanism of action similar to vancomycin. Teicoplanin retains activity against VRE carrying the *vanB* operon but not isolates with the *vanA* gene cluster. However, mutations resulting in constitutive activation of the *vanB* operon have led to teicoplanin failures. Teicoplanin is not approved by the FDA but is used in both Australia (approved in 1994) and the European Union (approved in 1988) at a dose of 6 mg/kg/day for cSSTIs, pneumonia, and complicated UTIs and 12 mg/kg/day for bone and joint infections and infective endocarditis caused by Gram-positive bacteria. Few studies have been conducted on the efficacy of teicoplanin against enterococcal infections, despite teicoplanin being used routinely for *vanB* VRE BSIs in Australia. In a retrospective review at two Australian healthcare centers (2008–2014) where *vanB* VRE is endemic, the effectiveness of teicoplanin monotherapy was analyzed (95). Teicoplanin (6–12 mg/kg) administered 12-hourly for three doses was associated with lower rates of ICU admission at 48 hours of VRE BSI (OR = 4.16; 95% CI = 1.08–16.00; *P* = 0.038), but did not influence mortality at 30 days (OR = 0.57; 95% CI = 0.20–1.64; *P* = 0.299) compared to other VRE-active agents such as linezolid and daptomycin or sequential therapy with any of these agents (95). In a prospective multicohort study in South Korea, patients with vancomycin-susceptible enterococcal BSIs were treated with either teicoplanin or vancomycin for ≥48 hours (96). There was no significant difference in 30-day mortality (*P* = 0.358) or 7-day mortality (*P* = 0.212) in patients treated with teicoplanin or vancomycin for vancomycin-susceptible *E. faecium* bacteremia. Thus, more clinical data are needed to add confidence in the utilization of teicoplanin for severe infections caused by *vanB*-carrying VRE.

## EMERGENCE OF THE PENICILLIN-RESISTANT, AMPICILLIN-SUSCEPTIBLE PHENOTYPE IN *ENTEROCOCCUS FAECALIS* AND IMPLICATIONS FOR THE EFFECTIVENESS OF THE AMPICILLIN-CEFTRIAXONE COMBINATION

The current standard of care for severe infections (including IE) caused by *E. faecalis* is the combination of ampicillin with ceftriaxone or ampicillin with gentamicin (or streptomycin). However, due to the toxicity of aminoglycosides, most clinicians are using the former combination as first-line therapy. The basis for this approach is the differential activity on PBPs of both drugs. Indeed, ampicillin has higher reactivity with PBP4 (a class B monofunctional transpeptidase), whereas ceftriaxone appears to have higher affinity for some bi-functional class A PBPs (PonA, PbpA, and PbpF) (97). This “dual” PBP effect is bactericidal and appears to be as efficacious as the combination of ampicillin plus gentamicin (98). However, in the last few years, several reports have described the emergence of reduced susceptibility of *E. faecalis* to penicillin, aminopenicillins, and piperacillin-tazobactam (99–101) associated with changes in the promoter of the gene encoding PBP4 or substitutions in the actual PBP that are likely to decrease the affinity of the PBP for β-lactam antibiotics. The emergence of the penicillin-resistant and ampicillin-susceptible phenotype in *E. faecalis* has been reported in clinical isolates from Brazil (102), South Korea (99), and Poland (103) and is associated with two major changes in PBP4. Some *in vitro* data suggest that these changes may diminish the activity of the ampicillin plus ceftriaxone combination, potentially leading to ineffective synergy *in vivo* (104). A prospective observational study suggested the 30-day mortality rates of patients with *E. faecalis* BSIs were twofold higher (26.9%; *n* = 18/67) in BSIs caused by penicillin-resistant strains than penicillin-susceptible isolates, likely due to treatment failures with piperacillin, where most MICs were ≥32 µg/mL (99). Moreover, an ampicillin non-susceptible strain of *E. faecalis* has emerged in Chile, harboring a deletion in the gene encoding PBP4, which may lead to a truncated PBP4 (105). However, western blot analysis showed no difference in PBP4 production compared to a wild-type laboratory strain. This finding is currently under active investigation (105). The role of the latest generation cephalosporins, such as ceftaroline and ceftobiprole, in the treatment of severe *E. faecalis* infections is unclear, although they are more potent than other cephalosporins and seem to have activity in combination with ampicillin in a retrospective case series (106, 107).

## PHAGE AS AN EMERGING TREATMENT OPTION

The dissemination of front- and last-line antimicrobial resistance in *E. faecium* and *E. faecalis* increases the need for developing alternative therapies, particularly for long-term DTR infections where effective treatment is vital. Bacteriophage (phage) therapy is an alternative to antibiotics and a newer therapeutic option for infections caused by multidrug-resistant bacteria, including VRE, with a Phase I/Phase IIa clinical trial to evaluate the safety and efficacy of phage therapy (VRElysin) ongoing in the United States (Intralytix). Several studies have isolated phages active against VRE, including phages that can infect strains resistant to linezolid and DAP (108, 109). The development of phage cocktails, where multiple phages are given to a patient, is of particular interest since *in vitro* experimental evidence suggests multiple phages are more effective at killing than a single phage, potentially due to the narrow host range. Phage cocktails are thought to decrease the chance that phage-resistant mutants will emerge, as such a development may require the simultaneous evolution of multiple resistance mechanisms. A mixture of 19 enterococcal phages was combined into different cocktails to test this hypothesis with VRE, where combinations of two or three phages were able to prevent the growth of phage-resistant VRE mutants that emerged against single phages (110). Strains of VRE that were phage-resistant consistently gained mutations in the exopolysaccharide synthesis genes (*epaE, epaR,* or *epaW*) present in the *Enterococcus* core genome. However, further genomic studies using a different panel of phages are needed to elucidate the full suite of resistance mechanisms and the baseline prevalence of these mutations in the VRE population.

Phages also offer a potential adjunctive therapy for patients who do not respond to antibiotic treatment alone. Pre-clinical studies have demonstrated a synergistic effect from combining phages with systemic antibiotics, with certain phages able to “rescue” DAP susceptibility in resistant *E. faecium* strains *in vitro*. In a patient with recurrent vancomycin-resistant *E. faecium* bacteremia, antibiotic combination therapy (DAP plus β-lactam or DAP plus tigecycline) was ineffective at long-term treatment since the patient was colonized with vancomycin-resistant *E. faecium,* and subsequent infections were emerging from the GIT (51). The addition of phage (Φ9184 and ΦHi3) to the patient’s antimicrobial therapy was able to successfully manage the BSI and reduce the abundance of VRE in the GIT. However, an anti-phage antibody response emerged (day 337) that neutralized the phage’s activity. The rational design of phage therapy with knowledge of phage-host interactions may advance phages as antibiotic alternatives or antibiotic adjuvants for the treatment of *Enterococcus* spp. Despite this initial promising data, clinical data to support phage use in DTR enterococcal infection are scarce.

## SUGGESTED APPROACH FOR DTR ENTEROCOCCAL INFECTIONS

**Fig 1:**
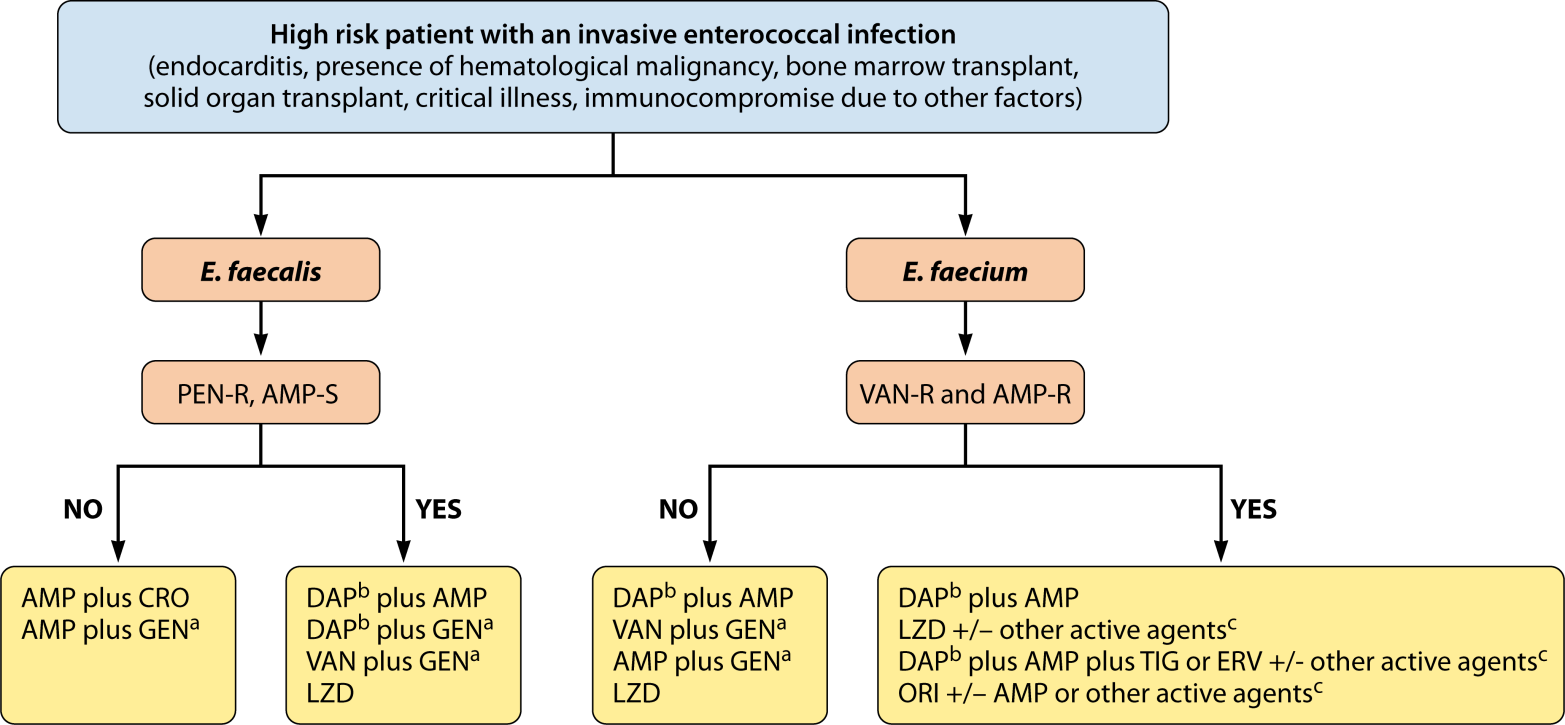
Suggested approach for difficult-to-treat enterococcal infections in high-risk patients (see text). PEN, penicillin; AMP, ampicillin; CRO, ceftriaxone; GEN, gentamicin; DAP, daptomycin; LZD, linezolid; ORI, oritavancin; TIG, tigecycline; ERV, eravacycline. ^a^If no high-level resistance is reported, streptomycin is also an option. Renal toxicity may limit the use of aminoglycosides, particularly when combined with VAN. ^b^Doses of at least 10 mg/kg should be used. ^c^Other agents with potential *in vitro* activity include aminoglycosides (GEN or streptomycin), oxazolidinones (LZD, tedizolid, contezolid), and tetracycline derivatives (TIG, ERV).

Difficult-to-treat enterococcal infections usually occur in debilitated patients with multiple co-morbidities, particularly in those with hematological malignancies or solid organ transplants. Infective enterococcal endocarditis (mostly caused by *E. faecalis*) is also challenging to treat, even though patients may not be as immunocompromised. In any case, these infections are considered high-risk for poor outcomes and should be aggressively treated (Fig. 1). In case of bacteremia and IE, prompt microbiological eradication seems of paramount importance to improve patients’ outcomes. Additionally, source control plays a major role in treating enterococcal infections and may prevent the development of resistance during therapy.

The standard of care for severe infections caused by *E. faecalis* continues to be the combination of ampicillin plus ceftriaxone (see rationale above). This combination is preferred by many since it is less prone to toxicity with apparent similar efficacy. However, the emergence of the penicillin-resistant, ampicillin-susceptible phenotype may jeopardize the effectiveness of the ampicillin-ceftriaxone combination (104). A good approach to identify this phenotype is to perform penicillin susceptibilities on isolates from deep-seated infections such as IE. If the organism is found to be resistant to penicillin (likely mediated by changes in or expression of PBP4/*pbp4*, see above), the ampicillin-ceftriaxone combination should be cautiously used, and consideration should be given to alternative treatments. The combination of DAP plus ampicillin is likely to be highly efficacious in these circumstances (Fig. 1). Indeed, we favor the combination over DAP monotherapy due to the ability of these organisms to develop DAP resistance during therapy, particularly since DAP resistance pathways overlap with those triggered by antimicrobial peptides produced by the immune system. DAP exposure is likely to select for resistant subpopulations that have adapted the cell envelopes to the antibiotic attack. The addition of ampicillin is likely to not only prevent the emergence of resistance but also increase the therapeutic efficacy of DAP via the seesaw effect. The caveat to this approach is the limited supporting clinical data and potential toxicities. Moreover, it is unclear if a combination would be required for the entire treatment period or can be “de-escalated” (e.g., DAP monotherapy), once the patient is stable and blood cultures have cleared.

For multidrug-resistant *E. faecium*, the most DTR of the enterococcal species, we favor initial empiric DAP combination therapy with a β-lactam (specifically ampicillin if possible despite phenotypic resistance to this antibiotic), while awaiting susceptibility testing and guided by local antimicrobial resistance data. This preference is based on the retrospective data suggesting better outcomes with high-dose DAP as compared to linezolid, and the *in vitro* data suggesting ampicillin both potentiates DAP activity and may prevent the emergence of resistance. Definitive therapy should be adjusted based on susceptibility data. Again, it is unclear whether the combination would be needed for the entire course of treatment, but it seems reasonable to maintain combination therapy if source control is not fully achieved. In case of intra-abdominal infections, particularly affecting the biliary tract, or in cases of persistent bacteremia in neutropenic patients where the source is thought to be related to disruptions in the GIT microbiome, the addition of eravacycline (or tigecycline) to the DAP plus ampicillin combination might be reasonable to achieve clearance of the blood stream (Fig. 1), although only anecdotal clinical data are available for this approach.

Linezolid monotherapy or in combination with other agents is also a possibility. However, as discussed above, linezolid does not show robust evidence of synergism with any particular anti-enterococcal agent, resistance can emerge with single mutations in the 23S rRNA genes, and the toxicity profile may limit its administration for prolonged periods of time. The rationale for adding linezolid or other agents is based on increasing activity against the infecting organisms and, possibly, impairing the ability to develop resistance.

An interesting phenomenon is the emergence of a “triple threat” in enterococci that includes resistance to ampicillin, vancomycin, and DAP. The antimicrobial choices are limited in this scenario. However, due to the see-saw effect, it seems reasonable to include the combination of DAP plus ampicillin, plus the addition of another active agent (e.g., linezolid, eravacycline/tigecycline, Fig. 1) as an option in high-risk patients. A caveat of this approach is that resistance mediated by pathways other than LiaFSR may not exhibit the see-saw effect, particularly in the setting of high MICs. An interesting alternative to treat this difficult multidrug-resistant infection is the use of oritavancin (see above). This lipoglycopeptide is active *in vitro* against DAP-resistant and vancomycin-resistant *E. faecium* and seems to also trigger the see-saw effect, suggesting that combination with ampicillin (94) may add therapeutic benefit. Oritavancin can also be combined with other active agents (Fig. 1). The major issue of the use of oritavancin is that the appropriate dosing scheme is not known when used in patient populations with severe VRE infections. In addition, clinical data to support oritavancin efficacy are also lacking.

## CONCLUDING REMARKS AND FUTURE DIRECTIONS

Multidrug-resistant enterococcal infections continue to be a major clinical challenge. The situation is compounded by the fact that clinical data are lacking to inform the best therapeutic approaches for these DTR infections. In the majority of cases, mechanistic insights supported by *in vitro* and *in vivo* data are the guiding principles to the therapeutic approach. Novel therapies that include phages, microbiome manipulation, and possibly, novel compounds with *in vitro* activity against these organisms are being tested, but most of these approaches are still far from becoming available for routine use. As such, there is an urgent need to gather clinical data to guide the best available therapies for these infections.

## References

[1] Sherry NL, Lee RS, Gorrie CL, Kwong JC, Stuart RL, Korman TM, Marshall C, Higgs C, Chan HT, Graham M, Johnson PDR, Leroi MJ, Reed C, Richards MJ, Slavin MA, Worth LJ, Howden BP, Grayson ML, Controlling Superbugs Study Group. 2021. Pilot study of a combined genomic and epidemiologic surveillance program for hospital-acquired multidrug-resistant pathogens across multiple hospital networks in Australia. Infect Control Hosp Epidemiol 42:573–581. doi:10.1017/ice.2020.1253 34008484

[2] Contreras GA, Munita JM, Simar S, Luterbach C, Dinh AQ, Rydell K, Sahasrabhojane PV, Rios R, Diaz L, Reyes K, et al.. 2022. Contemporary clinical and molecular epidemiology of vancomycin-resistant enterococcal bacteremia: a prospective multicenter cohort study (VENOUS I). Open Forum Infect Dis 9:ofab616. doi:10.1093/ofid/ofab616 35155713 PMC8830530

[3] Bradley CR, Fraise AP. 1996. Heat and chemical resistance of enterococci. J Hosp Infect 34:191–196. doi:10.1016/s0195-6701(96)90065-1 8923273

[4] Pidot SJ, Gao W, Buultjens AH, Monk IR, Guerillot R, Carter GP, Lee JYH, Lam MMC, Grayson ML, Ballard SA, Mahony AA, Grabsch EA, Kotsanas D, Korman TM, Coombs GW, Robinson JO, Gonçalves da Silva A, Seemann T, Howden BP, Johnson PDR, Stinear TP. 2018. Increasing tolerance of hospital Enterococcus faecium to handwash alcohols. Sci Transl Med 10:eaar6115. doi:10.1126/scitranslmed.aar6115 30068573

[5] Arias CA, Murray BE. 2012. The rise of the Enterococcus: beyond vancomycin resistance. Nat Rev Microbiol 10:266–278. doi:10.1038/nrmicro2761 22421879 PMC3621121

[6] García-Solache M, Rice LB. 2019. The Enterococcus: a model of adaptability to its environment. Clin Microbiol Rev 32:e00058-18. doi:10.1128/CMR.00058-18 30700430 PMC6431128

[7] Galloway-Peña J, Roh JH, Latorre M, Qin X, Murray BE. 2012. Genomic and SNP analyses demonstrate a distant separation of the hospital and community-associated clades of Enterococcus faecium. Plos One 7:e30187. doi:10.1371/journal.pone.0030187 22291916 PMC3266884

[8] Lebreton F, van Schaik W, McGuire AM, Godfrey P, Griggs A, Mazumdar V, Corander J, Cheng L, Saif S, Young S, Zeng Q, Wortman J, Birren B, Willems RJL, Earl AM, Gilmore MS. 2013. Emergence of epidemic multidrug-resistant Enterococcus faecium from animal and commensal strains. mBio 4:e00534-13. doi:10.1128/mBio.00534-13 23963180 PMC3747589

[9] Mills EG, Hewlett K, Smith AB, Griffith MP, Pless L, Sundermann AJ, Harrison LH, Zackular JP, Van Tyne D. 2025. Bacteriocin production facilitates nosocomial emergence of vancomycin-resistant Enterococcus faecium. Nat Microbiol 10:871–881. doi:10.1038/s41564-025-01958-0 40119148 PMC11964922

[10] Kaki R, Yu Y, O’Neill C, Lee C, Mertz D, Hamilton Health Sciences Infection Prevention and Control Team. 2014. Vancomycin-resistant enterococcus (VRE) transmission and risk factors in contacts of VRE carriers. Infect Control Hosp Epidemiol 35:876–879. doi:10.1086/676864 24915218

[11] Ziakas PD, Pliakos EE, Zervou FN, Knoll BM, Rice LB, Mylonakis E. 2014. MRSA and VRE colonization in solid organ transplantation: a meta-analysis of published studies. Am J Transplant 14:1887–1894. doi:10.1111/ajt.12784 25040438

[12] Alevizakos M, Gaitanidis A, Nasioudis D, Tori K, Flokas ME, Mylonakis E. 2017. Colonization with vancomycin-resistant enterococci and risk for bloodstream infection among patients with malignancy: a systematic review and meta-analysis. Open Forum Infect Dis 4:ofw246. doi:10.1093/ofid/ofw246 28480243 PMC5414102

[13] Ponce DM, Kosuri S, Khera N, DeFilipp Z, Lichter D, Lombardo M-J, Peled JU, van den Brink MRM, Chafee M, Wortman JR, Straub T, Walsh E, Ge A, Lyttle D, Brady K, Puhl MD, Glick G, Hasson BR, Tejura B, Ford CB, Henn MR, von Moltke L. 2024. Preliminary results of the open-label phase of a 2-part phase 1b study that evaluates safety, tolerability, pharmacokinetics, and efficacy of investigational microbiome therapeutic SER-155 in adults undergoing allogeneic Hematopoietic Cell Transplantation (allo-HCT). Transplantation and Cellular Therapy 30:S266. doi:10.1016/j.jtct.2023.12.355

[14] Shukla BS, Shelburne S, Reyes K, Kamboj M, Lewis JD, Rincon SL, Reyes J, Carvajal LP, Panesso D, Sifri CD, Zervos MJ, Pamer EG, Tran TT, Adachi J, Munita JM, Hasbun R, Arias CA. 2016. Influence of minimum inhibitory concentration in clinical outcomes of Enterococcus faecium bacteremia treated with daptomycin: is it time to change the breakpoint? Clin Infect Dis 62:1514–1520. doi:10.1093/cid/ciw173 27045126 PMC4885651

[15] Simar SR, Tran TT, Rydell KB, Atterstrom RL, Sahasrabhojane PV, Dinh AQ, Schettino MG, Slanis HS, Deyanov AE, DeTranaltes AM, et al.. 2025. Clinical and genomic characterization of recalcitrant enterococcal bacteremia: a multicenter prospective cohort study (VENOUS). J Infect Dis:jiaf358. doi:10.1093/infdis/jiaf358 40629152 PMC12718003

[16] Rogers R, Rice LB. 2024. State-of-the-art review: persistent enterococcal bacteremia. Clin Infect Dis 78:e1–e11. doi:10.1093/cid/ciad612 38018162

[17] Barie PS, Christou NV, Dellinger EP, Rout WR, Stone HH, Waymack JP. 1990. Pathogenicity of the Enterococcus in surgical infections. Ann Surg 212:155–159. doi:10.1097/00000658-199008000-00007 2198000 PMC1358050

[18] Ubeda C, Taur Y, Jenq RR, Equinda MJ, Son T, Samstein M, Viale A, Socci ND, van den Brink MRM, Kamboj M, Pamer EG. 2010. Vancomycin-resistant Enterococcus domination of intestinal microbiota is enabled by antibiotic treatment in mice and precedes bloodstream invasion in humans. J Clin Invest 120:4332–4341. doi:10.1172/JCI43918 21099116 PMC2993598

[19] Caballero S, Kim S, Carter RA, Leiner IM, Sušac B, Miller L, Kim GJ, Ling L, Pamer EG. 2017. Cooperating commensals restore colonization resistance to vancomycin-resistant Enterococcus faecium. Cell Host Microbe 21:592–602. doi:10.1016/j.chom.2017.04.002 28494240 PMC5494988

[20] Arias CA, Contreras GA, Murray BE. 2010. Management of multidrug-resistant enterococcal infections. Clin Microbiol Infect 16:555–562. doi:10.1111/j.1469-0691.2010.03214.x 20569266 PMC3686902

[21] Pericàs JM, Llopis J, Muñoz P, Gálvez-Acebal J, Kestler M, Valerio M, Hernández-Meneses M, Goenaga MÁ, Cobo-Belaustegui M, Montejo M, et al.. 2020. A contemporary picture of enterococcal endocarditis. J Am Coll Cardiol 75:482–494. doi:10.1016/j.jacc.2019.11.047 32029130

[22] Bussini L, Rosselli Del Turco E, Pasquini Z, Scolz K, Amedeo A, Beci G, Giglia M, Tedeschi S, Pascale R, Ambretti S, Pericàs JM, Giannella M, Carvalho-Brugger S, Gutiérrez L, Viale P, Bartoletti M. 2022. Risk factors for persistent enterococcal bacteraemia: a multicentre retrospective study. J Glob Antimicrob Resist 29:386–389. doi:10.1016/j.jgar.2022.05.003 35569757

[23] Grein F, Müller A, Scherer KM, Liu X, Ludwig KC, Klöckner A, Strach M, Sahl HG, Kubitscheck U, Schneider T. 2020. Ca(2+)-Daptomycin targets cell wall biosynthesis by forming a tripartite complex with undecaprenyl-coupled intermediates and membrane lipids. Nat Commun 11:1455. doi:10.1038/s41467-020-15257-1 32193379 PMC7081307

[24] Turnidge J, Kahlmeter G, Cantón R, MacGowan A, Giske CG. 2020. Daptomycin in the treatment of enterococcal bloodstream infections and endocarditis: a EUCAST position paper. Clin Microbiol Infect 26:1039–1043. doi:10.1016/j.cmi.2020.04.027 32353412

[25] Satlin MJ, Nicolau DP, Humphries RM, Kuti JL, Campeau SA, Lewis Ii JS, Weinstein MP, Jorgensen JH. 2020. Development of daptomycin susceptibility breakpoints for Enterococcus faecium and revision of the breakpoints for other enterococcal species by the clinical and laboratory standards institute. Clin Infect Dis 70:1240–1246. doi:10.1093/cid/ciz845 31504338

[26] Humphries RM. 2019. The new, new daptomycin breakpoint for Enterococcus spp. J Clin Microbiol 57. doi:10.1128/JCM.00600-19 PMC6595459 31092593

[27] Diaz L, Tran TT, Munita JM, Miller WR, Rincon S, Carvajal LP, Wollam A, Reyes J, Panesso D, Rojas NL, Shamoo Y, Murray BE, Weinstock GM, Arias CA. 2014. Whole-genome analyses of Enterococcus faecium isolates with diverse daptomycin MICs. Antimicrob Agents Chemother 58:4527–4534. doi:10.1128/AAC.02686-14 24867964 PMC4136017

[28] Miller WR, Nguyen A, Singh KV, Rizvi S, Khan A, Erickson SG, Egge SL, Cruz M, Dinh AQ, Diaz L, Thornton PC, Zhang R, Xu L, Garsin DA, Shamoo Y, Arias CA. 2025. Membrane lipids augment cell envelope stress signaling via the MadRS system to defend against antimicrobial peptides and antibiotics in Enterococcus faecalis. J Infect Dis 231:307–317. doi:10.1093/infdis/jiae173 38578967 PMC11841629

[29] Turner AM, Li L, Monk IR, Lee JYH, Ingle DJ, Portelli S, Sherry NL, Isles N, Seemann T, Sharkey LK, et al.. 2024. Rifaximin prophylaxis causes resistance to the last-resort antibiotic daptomycin. Nature 635:969–977. doi:10.1038/s41586-024-08095-4 39443798 PMC11602712

[30] Prater AG, Mehta HH, Kosgei AJ, Miller WR, Tran TT, Arias CA, Shamoo Y. 2019. Environment shapes the accessible daptomycin resistance mechanisms in Enterococcus faecium. Antimicrob Agents Chemother 63. doi:10.1128/AAC.00790-19 PMC6761497 31332078

[31] Arias CA, Panesso D, McGrath DM, Qin X, Mojica MF, Miller C, Diaz L, Tran TT, Rincon S, Barbu EM, Reyes J, Roh JH, Lobos E, Sodergren E, Pasqualini R, Arap W, Quinn JP, Shamoo Y, Murray BE, Weinstock GM. 2011. Genetic basis for in vivo daptomycin resistance in enterococci. N Engl J Med 365:892–900. doi:10.1056/NEJMoa1011138 21899450 PMC3205971

[32] Werth BJ, Steed ME, Ireland CE, Tran TT, Nonejuie P, Murray BE, Rose WE, Sakoulas G, Pogliano J, Arias CA, Rybak MJ. 2014. Defining daptomycin resistance prevention exposures in vancomycin-resistant Enterococcus faecium and E. faecalis. Antimicrob Agents Chemother 58:5253–5261. doi:10.1128/AAC.00098-14 24957825 PMC4135850

[33] Ramos Y, Sansone S, Hwang SM, Sandoval TA, Zhu M, Zhang G, Cubillos-Ruiz JR, Morales DK. 2022. Remodeling of the enterococcal cell envelope during surface penetration promotes intrinsic resistance to stress. mBio 13:e0229422. doi:10.1128/mbio.02294-22 36354750 PMC9765498

[34] DiPippo AJ, Tverdek FP, Tarrand JJ, Munita JM, Tran TT, Arias CA, Shelburne SA, Aitken SL. 2017. Daptomycin non-susceptible Enterococcus faecium in leukemia patients: role of prior daptomycin exposure. J Infect 74:243–247. doi:10.1016/j.jinf.2016.11.004 27845153 PMC5324836

[35] Kebriaei R, Stamper KC, Singh KV, Khan A, Rice SA, Dinh AQ, Tran TT, Murray BE, Arias CA, Rybak MJ. 2020. Mechanistic insights into the differential efficacy of daptomycin plus β-lactam combinations against daptomycin-resistant Enterococcus faecium. J Infect Dis 222:1531–1539. doi:10.1093/infdis/jiaa319 32514561 PMC7529040

[36] Khan A, Nguyen A, Panesso D, Vitrac H, Miller WR, Tran TT, Shamoo Y, Arthur M, Arias CA. 2019. 903. Resensitization to β-lactams in enterococci depends on penicillin-binding protein (PBP) mislocalization and is mediated by a single protein that modulates cell membrane (CM) adaptation to daptomycin (DAP). Open Forum Infect Dis 6:S28–S29. doi:10.1093/ofid/ofz359.062

[37] Chuang YC, Lin HY, Yang JL, Lin CY, Huang SH, Wang JT, Chen YC, Chang SC. 2022. Influence of daptomycin doses on the outcomes of VRE bloodstream infection treated with high-dose daptomycin. J Antimicrob Chemother 77:2278–2287. doi:10.1093/jac/dkac164 35639586

[38] Sinel C, Jaussaud C, Auzou M, Giard JC, Cattoir V. 2016. Mutant prevention concentrations of daptomycin for Enterococcus faecium clinical isolates. Int J Antimicrob Agents 48:449–452. doi:10.1016/j.ijantimicag.2016.07.006 27546218

[39] Lee IK, Sng YP, Li WF, Chen CL, Wang CC, Lin CC, Chen IL. 2022. Importance of daptomycin dosage on the clinical outcome in liver transplant recipients with vancomycin-resistant enterococci infection. J Chemother 34:367–374. doi:10.1080/1120009X.2022.2031470 35075978

[40] Kebriaei R, Rice SA, Singh KV, Stamper KC, Dinh AQ, Rios R, Diaz L, Murray BE, Munita JM, Tran TT, Arias CA, Rybak MJ. 2018. Influence of inoculum effect on the efficacy of daptomycin monotherapy and in combination with β-lactams against daptomycin-susceptible Enterococcus faecium harboring LiaSR substitutions. Antimicrob Agents Chemother 62:e00315-18. doi:10.1128/AAC.00315-18 29760141 PMC6105850

[41] Desbonnet C, Tait-Kamradt A, Garcia-Solache M, Dunman P, Coleman J, Arthur M, Rice LB. 2016. Involvement of the eukaryote-like kinase-phosphatase system and a protein that interacts with penicillin-binding protein 5 in emergence of cephalosporin resistance in cephalosporin-sensitive class a penicillin-binding protein mutants in Enterococcus faecium. mBio 7:e02188-15. doi:10.1128/mBio.02188-15 27048803 PMC4959515

[42] Khan A, Davlieva M, Panesso D, Rincon S, Miller WR, Diaz L, Reyes J, Cruz MR, Pemberton O, Nguyen AH, Siegel SD, Planet PJ, Narechania A, Latorre M, Rios R, Singh KV, Ton-That H, Garsin DA, Tran TT, Shamoo Y, Arias CA. 2019. Antimicrobial sensing coupled with cell membrane remodeling mediates antibiotic resistance and virulence in Enterococcus faecalis. Proc Natl Acad Sci USA 116:26925–26932. doi:10.1073/pnas.1916037116 31818937 PMC6936494

[43] Sakoulas G, Rose W, Nonejuie P, Olson J, Pogliano J, Humphries R, Nizet V. 2014. Ceftaroline restores daptomycin activity against daptomycin-nonsusceptible vancomycin-resistant Enterococcus faecium. Antimicrob Agents Chemother 58:1494–1500. doi:10.1128/AAC.02274-13 24366742 PMC3957885

[44] Smith JR, Barber KE, Raut A, Rybak MJ. 2015. β-Lactams enhance daptomycin activity against vancomycin-resistant Enterococcus faecalis and Enterococcus faecium in in vitro pharmacokinetic/pharmacodynamic models. Antimicrob Agents Chemother 59:2842–2848. doi:10.1128/AAC.00053-15 25753639 PMC4394769

[45] Chuang YC, Wang JT, Yang JL, Lin CY, Huang SH, Chen YC, Chang SC. 2022. The combination of daptomycin with β-lactam antibiotics is more effective than daptomycin alone for vancomycin-resistant Enterococcus faecium bloodstream infection. J Infect Public Health 15:1396–1402. doi:10.1016/j.jiph.2022.10.017 36371936

[46] La YJ, Kim YC. 2022. Successful treatment of vancomycin-resistant Enterococcus species bone and joint infection with daptomycin plus beta lactam agents. Infect Chemother 54:797–802. doi:10.3947/ic.2022.0106 36596688 PMC9840966

[47] Munita JM, Mishra NN, Alvarez D, Tran TT, Diaz L, Panesso D, Reyes J, Murray BE, Adachi JA, Bayer AS, Arias CA. 2014. Failure of high-dose daptomycin for bacteremia caused by daptomycin-susceptible Enterococcus faecium harboring LiaSR substitutions. Clin Infect Dis 59:1277–1280. doi:10.1093/cid/ciu642 25107294 PMC4271039

[48] Chuang YC, Tseng TC, Wang JT, Lin CY, Huang SH, Chen YC, Chang SC. 2022. Influence of daptomycin dose and fosfomycin susceptibility on outcome of vancomycin-resistant Enterococcus faecium bloodstream infections treated with daptomycin and fosfomycin combination. J Antimicrob Chemother 77:1436–1443. doi:10.1093/jac/dkac023 35141753

[49] Tseng TC, Chuang YC, Yang JL, Lin CY, Huang SH, Wang JT, Chen YC, Chang SC. 2023. The combination of daptomycin with fosfomycin is more effective than daptomycin alone in reducing mortality of vancomycin-resistant enterococcal bloodstream infections: a retrospective, comparative cohort study. Infect Dis Ther 12:589–606. doi:10.1007/s40121-022-00754-1 36629997 PMC9925660

[50] Shah S, McManus D, Topal JE. 2022. Combination therapy of chloramphenicol and daptomycin for the treatment of infective endocarditis secondary to multidrug resistant Enterococcus faecium. Hosp Pharm 57:345–348. doi:10.1177/00185787211032364 35615488 PMC9125124

[51] Stellfox ME, Fernandes C, Shields RK, Haidar G, Hughes Kramer K, Dembinski E, Mangalea MR, Arya G, Canfield GS, Duerkop BA, Van Tyne D. 2024. Bacteriophage and antibiotic combination therapy for recurrent Enterococcus faecium bacteremia. mBio 15:e0339623. doi:10.1128/mbio.03396-23 38353560 PMC10936196

[52] García-de-la-Mària C, Gasch O, García-Gonzalez J, Soy D, Shaw E, Ambrosioni J, Almela M, Pericàs JM, Tellez A, Falces C, Hernandez-Meneses M, Sandoval E, Quintana E, Vidal B, Tolosana JM, Fuster D, Llopis J, Pujol M, Moreno A, Marco F, Miró JM. 2018. The combination of daptomycin and fosfomycin has synergistic, potent, and rapid bactericidal activity against methicillin-resistant Staphylococcus aureus in a rabbit model of experimental endocarditis. Antimicrob Agents Chemother 62:e02633-17. doi:10.1128/AAC.02633-17 29610194 PMC5971606

[53] Pujol M, Miró JM, Shaw E, Aguado JM, San-Juan R, Puig-Asensio M, Pigrau C, Calbo E, Montejo M, Rodriguez-Álvarez R, et al.. 2021. Daptomycin plus fosfomycin versus daptomycin alone for methicillin-resistant Staphylococcus aureus bacteremia and endocarditis: a randomized clinical trial. Clin Infect Dis 72:1517–1525. doi:10.1093/cid/ciaa1081 32725216 PMC8096235

[54] Swaney SM, Aoki H, Ganoza MC, Shinabarger DL. 1998. The oxazolidinone linezolid inhibits initiation of protein synthesis in bacteria. Antimicrob Agents Chemother 42:3251–3255. doi:10.1128/AAC.42.12.3251 9835522 PMC106030

[55] Marshall SH, Donskey CJ, Hutton-Thomas R, Salata RA, Rice LB. 2002. Gene dosage and linezolid resistance in Enterococcus faecium and Enterococcus faecalis. Antimicrob Agents Chemother 46:3334–3336. doi:10.1128/AAC.46.10.3334-3336.2002 12234875 PMC128780

[56] Bender JK, Cattoir V, Hegstad K, Sadowy E, Coque TM, Westh H, Hammerum AM, Schaffer K, Burns K, Murchan S, Novais C, Freitas AR, Peixe L, Del Grosso M, Pantosti A, Werner G. 2018. Update on prevalence and mechanisms of resistance to linezolid, tigecycline and daptomycin in enterococci in Europe: Towards a common nomenclature. Drug Resist Updat 40:25–39. doi:10.1016/j.drup.2018.10.002 30447411

[57] Giessing AMB, Jensen SS, Rasmussen A, Hansen LH, Gondela A, Long K, Vester B, Kirpekar F. 2009. Identification of 8-methyladenosine as the modification catalyzed by the radical SAM methyltransferase Cfr that confers antibiotic resistance in bacteria. RNA 15:327–336. doi:10.1261/rna.1371409 19144912 PMC2648713

[58] Crowe-McAuliffe C, Murina V, Turnbull KJ, Huch S, Kasari M, Takada H, Nersisyan L, Sundsfjord A, Hegstad K, Atkinson GC, Pelechano V, Wilson DN, Hauryliuk V. 2022. Structural basis for PoxtA-mediated resistance to phenicol and oxazolidinone antibiotics. Nat Commun 13:1860. doi:10.1038/s41467-022-29274-9 35387982 PMC8987054

[59] Santimaleeworagun W, Changpradub D, Hemapanpairoa J, Thunyaharn S. 2021. Optimization of linezolid dosing regimens for treatment of vancomycin-resistant enterococci infection. Infect Chemother 53:503. doi:10.3947/ic.2021.0034 34405596 PMC8511381

[60] Rayner CR, Forrest A, Meagher AK, Birmingham MC, Schentag JJ. 2003. Clinical pharmacodynamics of linezolid in seriously ill patients treated in a compassionate use programme. Clin Pharmacokinet 42:1411–1423. doi:10.2165/00003088-200342150-00007 14674791

[61] Boak LM, Li J, Rayner CR, Nation RL. 2007. Pharmacokinetic/pharmacodynamic factors influencing emergence of resistance to linezolid in an in vitro model. Antimicrob Agents Chemother 51:1287–1292. doi:10.1128/AAC.01194-06 17242144 PMC1855482

[62] Huang ST, Yang JL, Lin CY, Huang SH, Wang JT, Chuang YC, Chen YC, Chang SC. 2023. Risk factors for mortality after linezolid treatment of vancomycin-resistant Enterococcus bloodstream infection. Int J Infect Dis 129:96–102. doi:10.1016/j.ijid.2023.01.035 36736576

[63] Lopez-Luis BA, Ponce-De-León A, Ortiz-Brizuela E, Lambraño-Castillo D, Leal-Vega FJ, Tovar-Calderón YE, Bobadilla-Del-Valle M, Sifuentes-Osornio J. 2022. Risk factors associated with failure of linezolid therapy in vancomycin-resistant Enterococcus faecium bacteremia: a retrospective cohort study in a referral center in Mexico. Microb Drug Resist 28:744–749. doi:10.1089/mdr.2021.0333 35333619

[64] Rani V, Aye NK, Saksena R, Dabi KC, Mannan MAU, Gaind R. 2024. Risk factors and outcome associated with the acquisition of MDR linezolid-resistant Enterococcus faecium: a report from tertiary care centre. Eur J Clin Microbiol Infect Dis 43:767–775. doi:10.1007/s10096-024-04784-0 38372832

[65] Olearo F, Both A, Belmar Campos C, Hilgarth H, Klupp EM, Hansen JL, Maurer FP, Christner M, Aepfelbacher M, Rohde H. 2021. Emergence of linezolid-resistance in vancomycin-resistant Enterococcus faecium ST117 associated with increased linezolid-consumption. Int J Med Microbiol 311:151477. doi:10.1016/j.ijmm.2021.151477 33524636

[66] Britt NS, Potter EM, Patel N, Steed ME. 2017. Effect of continuous and sequential therapy among veterans receiving daptomycin or linezolid for vancomycin-resistant Enterococcus faecium bacteremia. Antimicrob Agents Chemother 61:e02216-16. doi:10.1128/AAC.02216-16 28264856 PMC5404546

[67] Britt NS, Potter EM, Patel N, Steed ME. 2015. Comparison of the effectiveness and safety of linezolid and daptomycin in vancomycin-resistant enterococcal bloodstream infection: a national cohort study of Veterans Affairs patients. Clin Infect Dis 61:871–878. doi:10.1093/cid/civ444 26063715 PMC4551009

[68] Prokocimer P, Bien P, Deanda C, Pillar CM, Bartizal K. 2012. In vitro activity and microbiological efficacy of tedizolid (TR-700) against Gram-positive clinical isolates from a phase 2 study of oral tedizolid phosphate (TR-701) in patients with complicated skin and skin structure infections. Antimicrob Agents Chemother 56:4608–4613. doi:10.1128/AAC.00458-12 22687509 PMC3421864

[69] Silva-Del Toro SL, Greenwood-Quaintance KE, Patel R. 2016. In vitro activity of tedizolid against linezolid-resistant staphylococci and enterococci. Diagn Microbiol Infect Dis 85:102–104. doi:10.1016/j.diagmicrobio.2016.02.008 26971179 PMC8067670

[70] Singh KV, Arias CA, Murray BE. 2019. Efficacy of tedizolid against enterococci and staphylococci, including cfr(+) strains, in a mouse peritonitis model. Antimicrob Agents Chemother 63:e02627-18. doi:10.1128/AAC.02627-18 30670435 PMC6437489

[71] The European Committee on Antimicrobial Susceptibility Testing (EUCAST). 2025. Breakpoint Tables for Interpretation of MICs and Zone Diameters v. Available from: https://www.eucast.org/clinical_breakpoints

[72] CLSI. 2025. Performance standards for antimicrobial susceptibility testing. Vol. CLSI supplement M100. Clinical and Laboratory Standards Institute

[73] Merck & Co., Inc. 2019. Sivextro (tedizolid) [package insert]. U.S. Food and Drug Administration Website. Available from: https://www.accessdata.fda.gov/drugsatfda_docs/label/2014/205435s000lbl.pdf

[74] Pfizer Inc. 2025. Zyvox (linezolid) [package insert]. U.S. Food and Drug Administration Website. Available from: https://www.accessdata.fda.gov/drugsatfda_docs/label/2014/021130s032

[75] Rodríguez-Gascón A, Aguirre-Quiñonero A, Aspiazu MAS, Canut-Blasco A. 2021. Pharmacokinetic/pharmacodynamic analysis of tedizolid phosphate compared to linezolid for the treatment of infections caused by Gram-positive bacteria. Antibiotics (Basel) 10:755. doi:10.3390/antibiotics10070755 34206434 PMC8300700

[76] Hoy SM. 2021. Contezolid: first approval. Drugs (Abingdon Engl) 81:1587–1591. doi:10.1007/s40265-021-01576-0 PMC8536612 34365606

[77] Guo Y, Han R, Zhang G, Yang Q, Xue F, Li Y, Zhu D, Hu F. 2023. Setting of the tentative epidemiological cut-off values of contezolid for Staphylococcus aureus, Enterococcus faecalis, Enterococcus faecium, Streptococcus pneumoniae and Streptococcus agalactiae. J Antimicrob Chemother 78:1055–1058. doi:10.1093/jac/dkad045 36849586

[78] Li Y, Peng Y, Zhang N, Liu H, Mao J, Yan Y, Wang S, Yang G, Liu Y, Li J, Huang X. 2022. Assessing the emergence of resistance in vitro and in vivo: Linezolid combined with fosfomycin against fosfomycin-sensitive and resistant Enterococcus. Infect Drug Resist 15:4995–5010. doi:10.2147/IDR.S377848 36065277 PMC9440711

[79] Chi J, Li Y, Zhang N, Liu H, Chen Z, Li J, Huang X. 2023. Fosfomycin enhances the inhibition ability of linezolid against biofilms of vancomycin-resistant Enterococcus faecium in vitro. Infect Drug Resist 16:7707–7719. doi:10.2147/IDR.S428485 38144225 PMC10748582

[80] Hegstad K, Pöntinen AK, Bjørnholt JV, Quist-Paulsen E, Sundsfjord A. 2024. The first tigecycline resistant Enterococcus faecium in Norway was related to tigecycline exposure. J Glob Antimicrob Resist 36:112–115. doi:10.1016/j.jgar.2023.12.002 38122982

[81] Ding L, Yang Y, Zheng C, Sun G, Han R, Guo Y, Yin D, Wu S, Zhu D, Hu F. 2022. Activities of eravacycline, tedizolid, norvancomycin, nemonoxacin, ceftaroline, and comparators against 1,871 Staphylococcus and 1,068 Enterococcus species isolates from China: updated report of the CHINET study 2019. Microbiol Spectr 10:e0171522. doi:10.1128/spectrum.01715-22 36326536 PMC9769667

[82] Hawser S, Kothari N, Monti F, Morrissey I, Siegert S, Hodges T. 2023. In vitro activity of eravacycline and comparators against Gram-negative and Gram-positive bacterial isolates collected from patients globally between 2017 and 2020. J Glob Antimicrob Resist 33:304–320. doi:10.1016/j.jgar.2023.04.017 37207925

[83] Boukthir S, Dejoies L, Zouari A, Collet A, Potrel S, Auger G, Cattoir V. 2022. Corrigendum to ’In vitro activity of eravacycline and mechanisms of resistance in enterococci. Int J Antimicrob Agents 60:106694. doi:10.1016/j.ijantimicag.2022.106694 36437133

[84] U.S. Food and Drug Administration. 2005. Susceptibility Test Interpretive Criteria (STIC). Available from: http://www.fda.gov/STIC

[85] Singh KV, Arias CA, Murray BE. 2021. Efficacy of omadacycline against multidrug-resistant Enterococcus faecium strains in a mouse peritonitis model. Antimicrob Agents Chemother 65:e0070921. doi:10.1128/AAC.00709-21 34125596 PMC8370196

[86] Schutt AC, Bohm NM. 2009. Multidrug-resistant Enterococcus faecium endocarditis treated with combination tigecycline and high-dose daptomycin. Ann Pharmacother 43:2108–2112. doi:10.1345/aph.1M324 19887592

[87] Toke O, Cegelski L, Schaefer J. 2006. Peptide antibiotics in action: investigation of polypeptide chains in insoluble environments by rotational-echo double resonance. Biochim Biophys Acta 1758:1314–1329. doi:10.1016/j.bbamem.2006.02.031 16616889

[88] Belley A, McKay GA, Arhin FF, Sarmiento I, Beaulieu S, Fadhil I, Parr TR, Moeck G. 2010. Oritavancin disrupts membrane integrity of Staphylococcus aureus and vancomycin-resistant enterococci to effect rapid bacterial killing. Antimicrob Agents Chemother 54:5369–5371. doi:10.1128/AAC.00760-10 20876372 PMC2981232

[89] Carvalhaes CG, Sader HS, Streit JM, Castanheira M, Mendes RE. 2022. Activity of oritavancin against gram-positive pathogens causing bloodstream infections in the united states over 10 years: focus on drug-resistant enterococcal subsets (2010-2019). Antimicrob Agents Chemother 66:e0166721. doi:10.1128/AAC.01667-21 34807761 PMC8846398

[90] Pfaller MA, Mendes RE, Sader HS, Castanheira M, Carvalhaes CG. 2023. Oritavancin in vitro activity against Gram-positive organisms from European medical centers: a 10-year longitudinal overview from the SENTRY Antimicrobial Surveillance Program (2010-2019). J Chemother 35:689–699. doi:10.1080/1120009X.2023.2259673 37746914

[91] Mazzitelli M, Scaglione V, Cattarin L, Franchin E, Stano P, Paci L, Coppi M, Rossolini GM, Mengato D, Calò L, Cattelan AM. 2024. Off-label oritavancin treatment outcome and molecular characterization of a vancomycin- and linezolid-resistant Enterococcus faecium causing liver abscesses. J Antimicrob Chemother 79:689–691. doi:10.1093/jac/dkad410 38225167

[92] Texidor WM, Miller MA, Molina KC, Krsak M, Calvert B, Hart C, Storer M, Fish DN. 2024. Oritavancin as sequential therapy for Gram-positive bloodstream infections. BMC Infect Dis 24:127. doi:10.1186/s12879-023-08725-8 38267844 PMC10807122

[93] Giuliano G, Benedetti S, Sambo M, Pierguidi F, Tumbarello M. 2024. Successful treatment of complicated infective endocarditis due to Enterococcus faecium in a patient with substance use disorder using oritavancin as sequential maintenance therapy. Clin Microbiol Infect 30:556–557. doi:10.1016/j.cmi.2024.01.008 38253314

[94] Smith JR, Yim J, Raut A, Rybak MJ. 2016. Oritavancin combinations with β-lactams against multidrug-resistant Staphylococcus aureus and vancomycin-resistant enterococci. Antimicrob Agents Chemother 60:2352–2358. doi:10.1128/AAC.03006-15 26833159 PMC4808215

[95] Xie O, Slavin MA, Teh BW, Bajel A, Douglas AP, Worth LJ. 2020. Epidemiology, treatment and outcomes of bloodstream infection due to vancomycin-resistant enterococci in cancer patients in a vanB endemic setting. BMC Infect Dis 20:228. doi:10.1186/s12879-020-04952-5 32188401 PMC7079500

[96] Ha S, Huh K, Chung DR, Ko J-H, Cho SY, Huh HJ, Lee NY, Kang C-I, Peck KR, Song J-H, Korean Antimicrobial Resistance Surveillance Network (KARS-Net) investigators. 2022. Efficacy of teicoplanin in bloodstream infections caused by Enterococcus faecium: posthoc analysis of a nationwide surveillance. Int J Infect Dis 122:506–513. doi:10.1016/j.ijid.2022.06.029 35752376

[97] Djorić D, Little JL, Kristich CJ. 2020. Multiple low-reactivity class B penicillin-binding proteins are required for cephalosporin resistance in enterococci. Antimicrob Agents Chemother 64:e02273-19. doi:10.1128/AAC.02273-19 32041714 PMC7179317

[98] Fernández-Hidalgo N, Almirante B, Gavaldà J, Gurgui M, Peña C, de Alarcón A, Ruiz J, Vilacosta I, Montejo M, Vallejo N, López-Medrano F, Plata A, López J, Hidalgo-Tenorio C, Gálvez J, Sáez C, Lomas JM, Falcone M, de la Torre J, Martínez-Lacasa X, Pahissa A. 2013. Ampicillin plus ceftriaxone is as effective as ampicillin plus gentamicin for treating Enterococcus faecalis infective endocarditis. Clin Infect Dis 56:1261–1268. doi:10.1093/cid/cit052 23392394

[99] Kim D, Lee H, Yoon EJ, Hong JS, Shin JH, Uh Y, Shin KS, Shin JH, Kim YA, Park YS, Jeong SH. 2019. Prospective observational study of the clinical prognoses of patients with bloodstream infections caused by ampicillin-susceptible but penicillin-resistant Enterococcus faecalis. Antimicrob Agents Chemother 63. doi:10.1128/AAC.00291-19 PMC6591605 31010856

[100] Metzidie E, Manolis EN, Pournaras S, Sofianou D, Tsakris A. 2006. Spread of an unusual penicillin- and imipenem-resistant but ampicillin-susceptible phenotype among Enterococcus faecalis clinical isolates. J Antimicrob Chemother 57:158–160. doi:10.1093/jac/dki427 16308417

[101] Conceição N, da Silva LEP, Darini AL da C, Pitondo-Silva A, de Oliveira AG. 2014. Penicillin-resistant, ampicillin-susceptible Enterococcus faecalis of hospital origin: pbp4 gene polymorphism and genetic diversity. Infect Genet Evol 28:289–295. doi:10.1016/j.meegid.2014.10.018 25445645

[102] Infante VHP, Conceição N, de Oliveira AG, Darini AL da C. 2016. Evaluation of polymorphisms in pbp4 gene and genetic diversity in penicillin-resistant, ampicillin-susceptible Enterococcus faecalis from hospitals in different states in Brazil. FEMS Microbiol Lett 363:fnw044. doi:10.1093/femsle/fnw044 26903013

[103] Gawryszewska I, Żabicka D, Hryniewicz W, Sadowy E. 2021. Penicillin-resistant, ampicillin-susceptible Enterococcus faecalis in Polish Hospitals. Microb Drug Resist 27:291–300. doi:10.1089/mdr.2019.0504 32640911

[104] Westbrook KJ, Chilambi GS, Stellfox ME, Nordstrom HR, Li Y, Iovleva A, Shah NH, Jones CE, Kline EG, Squires KM, Miller WR, Tran TT, Arias CA, Doi Y, Shields RK, Van Tyne D. 2024. Differential in vitro susceptibility to ampicillin/ceftriaxone combination therapy among Enterococcus faecalis infective endocarditis clinical isolates. J Antimicrob Chemother 79:801–809. doi:10.1093/jac/dkae032 38334390 PMC10984950

[105] Diaz L, Singh KV, Tran CT, Quiroz VE, Soto K, Peters AS, Paula Baptista R, Martinez JRW, Ugalde J, Hoppe G, Araos R, Acuna MP, Martinez I, Garcia P, Miller WR, Munita JM, Arias CA, Panesso D. 2025. Multi-hospital dissemination of ampicillin resistance associated with a frame-shift deletion in pbp4 of vancomycin-resistant Enterococcus faecalis. Open Forum Infect Dis 12:ofae631.1526. doi:10.1093/ofid/ofae631.1526

[106] Giuliano S, Angelini J, Campanile F, Conti P, Flammini S, Pagotto A, Sbrana F, Martini L, D’Elia D, Abdul-Aziz MH, Cotta MO, Roberts JA, Bonomo RA, Tascini C. 2025. Evaluation of ampicillin plus ceftobiprole combination therapy in treating Enterococcus faecalis infective endocarditis and bloodstream infection. Sci Rep 15:3519. doi:10.1038/s41598-025-87512-8 39875507 PMC11775251

[107] Giuliano S, Angelini J, D’Elia D, Geminiani M, Barison RD, Giacinta A, Sartor A, Campanile F, Curcio F, Cotta MO, Roberts JA, Baraldo M, Tascini C. 2023. Ampicillin and ceftobiprole combination for the treatment of Enterococcus faecalis invasive infections: “The Times They Are A-Changin”. Antibiotics (Basel) 12:879. doi:10.3390/antibiotics12050879 37237782 PMC10215339

[108] Qu Q, Chen T, He P, Geng H, Zeng P, Luan G. 2023. Isolation and characterization of a novel lytic bacteriophage vB_Efm_LG62 infecting Enterococcus faecium. Virus Genes 59:763–774. doi:10.1007/s11262-023-02016-9 37422898

[109] Pradal I, Casado A, del Rio B, Rodriguez-Lucas C, Fernandez M, Alvarez MA, Ladero V. 2023. Enterococcus faecium bacteriophage vB_EfaH_163, a new member of the herelleviridae family, reduces the mortality associated with an E. faecium vanR clinical isolate in a Galleria mellonella animal model. Viruses 15:179. doi:10.3390/v15010179 36680219 PMC9860891

[110] Wandro S, Ghatbale P, Attai H, Hendrickson C, Samillano C, Suh J, Dunham SJB, Pride DT, Whiteson K. 2022. Phage cocktails constrain the growth of Enterococcus. mSystems 7:e0001922. doi:10.1128/msystems.00019-22 35762793 PMC9426582

